# Visits to Private Asylums

**Published:** 1903-05-23

**Authors:** 


### VISITS TO PRIVATE ASYLUMS.

By Our Rambling Reporter.

### SPRINGFIELD, BEDFORD.

About an hour's run on the Midland line brings one from St. Pancras to Bedford, a very pleasant little town of 30,000 inhabitants, and long famous for its numerous schools. Twenty minutes' drive from the railway station stands the Springfield Private Asylum, a mansion in the late Georgian style of architecture, built specially for an asylum, and opened and licensed in the year 1837. The number it is licensed for is 48. It is surrounded by 40 acres of land, some of which is used for agricultural purposes; but much of it is laid out in pleasure grounds and lawns, and broad shady paths meander through the grounds, affording a walk of about one mile and a-half in length.

The brightness and the cheerful appearance of the rooms now noticed in so many of our private asylums is here presented to us in a very high degree. The sitting-rooms are veritable drawing-rooms and studies, and the bedrooms are exceptionally good. There is one dormitory for five beds, but the large majority of the patients have rooms of their own, and in most cases these rooms are above the average size found in asylums. They are also nicely decorated and furnished, are well warmed and efficiently ventilated by Tobin's tubes and Boyle's extractors. With one or two exceptions the bedrooms face either south or west. There is an absence of barred windows and prison-like arrangements of any kind, and everything has been done by Dr. Bower to give the entire asylum a home-like appearance. This effect has been obtained, and it is emphasised by the fact that the lady and gentlemen patients take their meals in the same dining-room. The improvements which have been made since Dr. Bower and his partner Miss Norton acquired the license have literally been enormous, and the institution has been worked up to a high standard, taking its place as one of the best private asylums of its class in England. Perfect order and cleanliness reign throughout the whole establishment. The precautions taken against the occurrence or spread of fire are good, and they include special escape staircases leading from the first floor direct to the outside, and as the building is only two stories in height there ought to be no danger of fatal accidents.

One of the greatest difficulties experienced in private asylums is to find suitable occupation for the patients, and yet it is certain that no treatment yet discovered equals healthy employment as a means of cure. This is one of the points that Dr. Bower has made a special study of, and something more than a beginning has been made at Springfield to solve the difficulty. Parties of the gentlemen patients find suitable occupation and recreation in the varied operations of farming and gardening, and we were satisfied that those so employing themselves were interested in their work and willingly took to it. The indoor and outdoor amusements are numerous. There are country drives almost daily, dances often take place, and billiards, golf, cricket, and other games fill up the time. Idleness is discouraged, and every patient is induced to do something.

One innovation made by Dr. Bower deserves to be specially referred to here, and we think it ought to be followed in some other of our private asylums. That is, there is only one rate of board for patients, and that rate is three guineas a week. By thus equalising payments invidious distinctions are removed from amid the patients, and all the members of the family under the Springfield roof are on the same footing. It is true that private sitting-rooms can be had, but it is an arrangement not recommended by Dr. Bower, which proves that his anxiety for the welfare of all his patients is greater than his own pecuniary interest, for, of course, by encouraging the use of these private rooms higher sums would be obtained. It is as well to say that equalising the payments by no means prevents the proper classification of the patients in relation to the forms of insanity under which they may be suffering.

There are on the grounds a few villas in which single patients can be accommodated.

The asylum staff averages about one attendant or nurse to three patients, and there are lady-companions in addition to this. The percentage of recoveries is high, standing between 50 and 60 per cent. of the admissions, and the death-rate is very low.

In our introductory article of December 20th, 1902, we stated our opinion that some of the private asylums should have the numbers they are licensed for increased. Springfield is pre-eminently one of these, and by its position (within 50 miles of London), its moderate rate of board, and its enlightened management it should not stop short of 100 beds.

